# Evaluating the Influence of Health Education Programs on Quality of Life in Patients with Type 2 Diabetes Mellitus in Saudi Arabia

**DOI:** 10.5812/ijem-167044

**Published:** 2025-10-31

**Authors:** Raneem Salem, Ayesha Nuzhat, Ghadeer Hassonah, Fatimah Al Barqi, Asirvatham Alwin Robert

**Affiliations:** 1 King Fahd Medical City, Riyadh Second Health Cluster, Riyadh, Saudi Arabia; 2 College of Medicine, Imam Mohammad Ibn Saud Islamic University, Riyadh, Saudi Arabia; 3 Department of Endocrinology and Diabetes, Prince Sultan Military Medical Center, Riyadh, Saudi Arabia; 4 Department of Health Education, King Fahd Medical City, Riyadh Second Health Cluster, Riyadh, Saudi Arabia

**Keywords:** Type 2 Diabetes Mellitus, Quality of Life, Health Education, Saudi Arabia, WHOQOL, Patient Awareness, Chronic Disease Management

## Abstract

**Background and Objective:**

The prevalence of type 2 diabetes in Saudi Arabia is steadily rising, resulting in multiple complications that negatively influence patients’ quality of life (QOL) and further add to the worldwide burden of chronic diseases. This study aimed to evaluate the QOL of individuals with diabetes and to determine the effect of structured health education programs on improving their QOL.

**Methods:**

A quasi-experimental study was carried out after obtaining ethical approval. A total of 232 diabetic patients were recruited from King Fahad Medical city and Prince Sultan Military Medical city. During the initial phase, sociodemographic and baseline clinical data were collected. Each patient then participated in 20 - 30-minute health education sessions every two weeks for a period of three months. After the intervention, follow-up assessments were performed using the same World Health Organization Quality of Life (WHOQOL) questionnaire to measure changes in QOL. Data were analyzed using Microsoft Excel for descriptive statistics, frequency distributions, and cross-tabulations, while Student’s *t*-test was applied to assess statistical significance, with P < 0.05 considered significant.

**Results:**

Among the participants, 117 (51%) were male and 115 (49%) were female; 25 (11%) were under 40 years old, while 207 (89%) were above 40 years. Most patients (198; 85%) had been receiving antidiabetic treatment for more than 10 years, whereas 44 (15%) had less than 10 years of treatment history. The average random blood sugar at baseline was 8.3 mmol/L. 206 (89%) patients were compliant to treatment and 216 (93%) had complications of diabetes. Post-intervention findings showed improvements across all four WHOQOL domains, with notable gains in the physical and psychological domains, and modest improvements in social and environmental aspects. Statistical analysis confirmed a significant difference between baseline and post-intervention scores in all domains (P < 0.05).

**Conclusions:**

Structured health education significantly enhances the QOL of diabetic patients across multiple domains. Strengthening patient awareness and self-care not only helps prevent serious complications and premature mortality but also reduces the burden on families, healthcare institutions, and the broader healthcare system.

## 1. Background

Globally, the prevalence of diabetes has continued to rise across all nations, as reported by the World Health Organization (WHO) (1-3). The Kingdom of Saudi Arabia is one of the 20 countries comprising the Middle East and North Africa (MENA) region. According to the International Diabetes Federation (IDF) Diabetes Atlas 2025, diabetes has emerged as a major global health challenge. An estimated 589 million adults between the ages of 20 and 79 years are currently living with the disease, representing one in every nine individuals worldwide. Projections indicate that this figure will rise dramatically, reaching 853 million by 2050, underscoring the accelerating pace at which diabetes is spreading across populations (4).

Saudi Arabia has experienced a particularly dramatic increase in the prevalence of diabetes mellitus (DM) over the past three decades, with nearly a tenfold rise in diagnosed cases (5-8). Recent Worldometer statistics indicate that, out of the nation’s 34.8 million residents, approximately 4.27 million have been diagnosed with diabetes, while an additional 1.86 million remain undiagnosed. Furthermore, the IDF projects that the prevalence of diabetes in Saudi Arabia will double by 2045 if current trends persist (9).

This rapid rise in diabetes prevalence, along with its associated complications, has significant implications for both individual quality of life (QOL) and the national healthcare system (10). In another study in 2014 using the Audit of Diabetes-Dependent Quality of Life (ADDQOL) Questionnaire found that 78.7% of Saudi patients with diabetes reported unfavorable QOL scores (11). Poorer outcomes were observed particularly among patients with type 2 diabetes and those with uncontrolled disease, with retinopathy, diabetic foot, and neuropathy identified as the complications most strongly associated with reduced QOL (12). Structured health education and diabetes self-management education interventions have been shown to significantly improve QOL and medication adherence among patients with type 2 diabetes mellitus (T2DM) (13).

Uncontrolled diabetes is well known to cause multi-system complications that substantially diminish QOL (14). Nevertheless, these adverse outcomes can often be prevented or mitigated through regular, structured health education focusing on disease awareness, glycemic control, and lifestyle modification (15). Such interventions are both practical and sustainable, as they can be delivered in outpatient settings by trained health educators (11). Systematically delivered health education holds considerable potential to enhance QOL across all domains: Physical, psychological, social, and environmental. While previous studies in Saudi Arabia have examined disease awareness, diabetes-related complications, and the quality of healthcare services (10, 11, 13), none have directly evaluated the impact of structured health education interventions on QOL outcomes. The present study was therefore designed to address this gap. Its objectives were twofold: First, to assess the baseline QOL among patients with diabetes, and second, to evaluate the effectiveness of structured health education interventions in improving their overall QOL.

## 2. Objectives

The present study was designed to address this gap. Its objectives were twofold: First, to assess the baseline QOL among patients with diabetes, and second, to evaluate the effectiveness of structured health education interventions in improving their overall QOL.

## 3. Methods

### 3.1. Study Design and Setting

This quasi-experimental study was conducted over a 10-month period (October 2022 - March 2023) at two tertiary care hospitals in Saudi Arabia: King Fahad Medical city and Prince Sultan Military Medical city. The study was approved by the Institutional Review Board (IRB No. 22-447) and conducted in accordance with the Declaration of Helsinki. Written informed consent was obtained from all participants. Data confidentiality was strictly maintained and accessible only to the research team. Although the intervention lasted three months, enough time was needed to compile the data from two hospitals and conduct data analysis, prepare the draft and review, followed by obtaining approval for the report by our institutional IRB before final submission.

### 3.2. Study Population

Patients with type 2 diabetes constituted the study population. The required sample size was calculated using RaoSoft software (16). At first, the estimated sample size was 377 (that was rounded off to 400) patients using a margin of error of 5%, 95% confidence interval (CI), population size 20000, and with a response distribution of 50%, and we decided to collect 200 from each hospital, King Fahad Medical city and Prince Sultan Military Medical city.

The estimated sample size was 195 patients after recalculation using a margin of error of 7%, 95% CI, population size 20000, and with a response distribution of 50%, and this sample size would suffice to give us significant results as an acceptable margin of error typically falls between 4% and 8% at the 95% CI (17).

We could finally acquire data from 232 patients, and could not collect data from the remaining, especially after intervention, due to a sudden change from face-to-face intervention to virtual intervention (health education of diabetes patients) in King Fahad Medical city.

### 3.3. Eligibility Criteria

Inclusion criteria were adults of either sex, aged 18 - 70 years, with a confirmed diagnosis of T2DM (HbA1c > 6.5) for more than two years through the records. Patients were excluded if they had chronic painful conditions (e.g., cancer, spinal injury), psychiatric disorders, or incomplete medical records.

### 3.4. Data Collection

Data collection was carried out in three sequential stages. During the pre-intervention phase, baseline information was obtained using a structured form that included sociodemographic characteristics, diabetes history, symptoms, comorbidities, treatment adherence, and diabetes-related complications. Patients’ QOL was assessed using the World Health Organization Quality of Life-BREF (WHOQOL-BREF) Questionnaire, a validated 24-item instrument covering four domains: Physical health (7 items), psychological well-being (6 items), social relationships (3 items), and environmental factors (8 items). Anthropometric measurements, including height and weight, were recorded to calculate Body Mass Index (BMI) using the standard formula [weight (kg)/height (m²)], and laboratory investigations included random blood glucose and glycated hemoglobin (HbA1c) (18, 19).

In the intervention phase, patients participated in structured virtual health education sessions conducted every two weeks over a three-month period. They interacted with the single researcher in each respective hospital of our research team. The majority of the patients were literate and were able to answer the questionnaire. If they were illiterate, the questionnaire was read out to them and their responses were noted by the researcher of our team working in the respective hospital. The researchers were qualified specialists working in the Diabetes and Health Education Department with more than 5 years of experience. They followed a standard protocol from the hospital based on Saudi Diabetes National Center guidelines (20).

Each session conducted by the researcher lasted 20 - 30 minutes and focused on the definition, risk factors, clinical features, and complications of diabetes, as well as treatment strategies, self-care practices including diet, exercise, and medication adherence, and psychosocial support.

The post-intervention phase was conducted three months after the completion of the educational program. During this stage, patients attended in-person follow-up visits, and the WHOQOL-BREF questionnaire was re-administered to evaluate changes in QOL across all domains (18).

### 3.5. Statistical Analysis

Data analysis was performed using Microsoft Excel 2019 (Microsoft Corporation, Redmond, WA, USA) and SPSS version 22.0 (SPSS Inc., Chicago, IL, USA). Continuous variables were presented as means ± standard deviations (SD), while categorical variables were summarized as frequencies and percentages. Descriptive statistics summarizing sociodemographic and clinical characteristics were done using Excel. Frequency distributions and cross-tabulations were performed for categorical variables. Comparisons of QOL scores between baseline and post-intervention stages were assessed using Student’s *t*-test using SPSS. A P-value of < 0.05 was considered statistically significant.

This study follows a prospective longitudinal design or pre-post study design utilizing a questionnaire-based approach. In studies with larger sample sizes > 200 participants, it is often challenging to retain and collect follow-up data from the same individuals across different time points. As a result, complete paired data may not be available for all participants. Given this limitation and to ensure robust statistical analysis despite potential loss to follow-up, it is reasonable and statistically appropriate to apply the independent sample *t*-test if matching responses are not assured. However, the questionnaire is structured to be administered at both the baseline and three months, aiming to capture changes over time wherever possible.

## 4. Results

Table 1 presents the baseline sociodemographic profile of the diabetic patients enrolled in the study. A total of 232 patients completed the study. The majority of participants were over 40 years of age (207, 89%), with 25 patients (11%) under 40 years, and the majority of them were literate (82%). The gender distribution was nearly equal, with 117 males (51%) and 115 females (49%). Most patients had a BMI above 25 kg/m² (202, 87%), and the majority had a long duration of diabetes, with 198 patients (85%) receiving treatment for more than 10 years. Regarding treatment modalities, 130 patients (56%) were on combined therapy (oral hypoglycemics plus insulin), 57 patients (25%) received insulin alone, 43 patients (19%) were on oral hypoglycemic agents only, and 2 patients (1%) were managing their diabetes through diet alone. The average random blood sugar at baseline was 8.3 mmol/L. A total of 206 (89%) patients were compliant to treatment and 216 (93%) had complications of diabetes.

**Table 1.** Sociodemographic Features of the Diabetic Patients

Variables	Values
**Age**	
< 40	25 (11)
> 40	207 (89)
**Gender**	
Male	117 (51)
Female	115 (49)
**Education**	
Illiterate	41 (18)
Literate	191 (82)
**BMI**	
< 25	30 (13)
> 25	202 (87)
**Duration of treatment**	
< 10	44 (14)
> 10	198 (85)
**Type of treatment**	
Oral	43 (19)
Insulin	57 (25)
Combined	130 (56)
Diet	2 (1)
**Compliance to treatment**	
Yes	206 (89)
No	26 (11)
**Complications**	
Yes	216 (93)
No	16 (7)

Table 2 shows the comparison of quality-of-life domain scores (physical, psychological, social, environmental) at baseline and 3 months across patient characteristics. Across all participants, scores in the physical, psychological, social, and environmental domains improved from baseline to 3 months. Physical health showed a clear rise in both age groups, especially among participants > 40 years, who demonstrated greater improvement than those ≤ 40 years. Psychological scores improved in most groups, with the highest gains seen in females, individuals with BMI ≥ 25, and those with longer diabetes duration (> 10 years). Social domain scores increased slightly across all categories, with consistent improvements in both gender and BMI groups. Environmental scores also improved from baseline, particularly among participants > 40 years, females, those with higher BMI, and those who were compliant with treatment. Overall, patients without complications showed better psychological and physical improvement compared to those with complications.

**Table 2.** Comparison of Quality-of-Life Domain Scores (Physical, Psychological, Social, Environmental) at Baseline and 3 Months Across Patient Characteristics

Variables	Physical		Psychological		Social		Environmental	
	Baseline	3 Months	Baseline	3 Months	Baseline	3 Months	Baseline	3 Months
**Age**								
≤ 40	22.2 ± 3.8	25.2 ± 4.6 ^a^	22.3 ± 5.0	24.1 ± 3.9	9.67 ± 2.8	9.25 ± 2.6	30.6 ± 7.0	27.3 ± 5.0
> 40	21.4 ± 3.5	27.1 ± 3.1 ^a^	19.3 ± 3.4	24.5 ± 2.2 ^a^	9.63 ± 2.1	10.4 ± 1.9	26.1 ± 5.4	28.7 ± 3.3 ^a^
**Gender**								
Male	21.5 ± 3.7	26.5 ± 3.5 ^a^	19.7 ± 4.0	24.4 ± 2.3 ^a^	9.8 ± 2.2	10.3 ± 2.0 ^a^	27.2 ± 6.2	28.4 ± 3.5 ^a^
Female	21.5 ± 3.4	27.1 ± 3.1 ^a^	19.7 ± 3.5	24.4 ± 2.6 ^a^	9.4 ± 2.1	10.1 ± 2.1	26.1 ± 5.3	28.7 ± 3.6 ^a^
**BMI**								
≤ 25	21.4 ± 3.9	26.5 ± 4.2 ^a^	19.4 ± 4.1	24.2 ± 2.8 ^a^	10.2 ± 2.3	10.3 ± 2.1	26.9 ± 5.5	28.6 ± 3.4 ^a^
> 25	21.5 ± 3.4	26.9 ± 3.1 ^a^	19.8 ± 3.6	24.5 ± 2.3 ^a^	9.4 ± 2.2	10.2 ± 2.0	26.5 ± 5.8	28.5 ± 3.7 ^a^
**Duration of diabetes (y)**								
≤ 10	21.4 ± 3.8	26.4 ± 3.1 ^a^	20.1 ± 4.1	24.4 ± 2.7 ^a^	9.0 ± 2.4	10.1 ± 1.7 ^a^	28.0 ± 6.2	28.6 ± 3.9
> 10	21.5 ± 3.5	26.9 ± 3.3 ^a^	19.6 ± 3.7	24.4 ± 2.4 ^a^	9.7 ± 2.1	10.2 ± 2.1 ^a^	26.4 ± 5.7	28.5 ± 3.5 ^a^
**Compliance to treatment**								
Yes	21.4 ± 3.6	27.2 ± 2.7 ^a^	19.4 ± 3.5	24.4 ± 2.3 ^a^	9.61 ± 2.1	10.3 ± 2.0	26.2 ± 5.5	28.6 ± 3.4 ^a^
No	22.3 ± 3.6	26.1 ± 4.7 ^a^	23.6 ± 4.3	24.6 ± 3.8	10.1 ± 2.0	9.37 ± 2.7	32.4 ± 6.4	27.1 ± 5.6
**Complications of diabetes**								
Yes	21.5 ± 3.6	27.1 ± 3.0 ^a^	19.4 ± 3.5	24.4 ± 2.3 ^a^	9.61 ± 2.1	10.3 ± 2.1 ^a^	26.2 ± 5.5	28.6 ± 3.4 ^a^
No	21.8 ± 3.4	23.8 ± 5.1	23.6 ± 4.3	24.6 ± 3.8	10 ± 2.6	9.37 ± 2.7	32.4 ± 6.4	27.1 ± 5.6

Abbreviation: BMI, Body Mass Index.

^a^ Indicates a statistically significant difference between baseline and 3 months.

**Figure 1.**
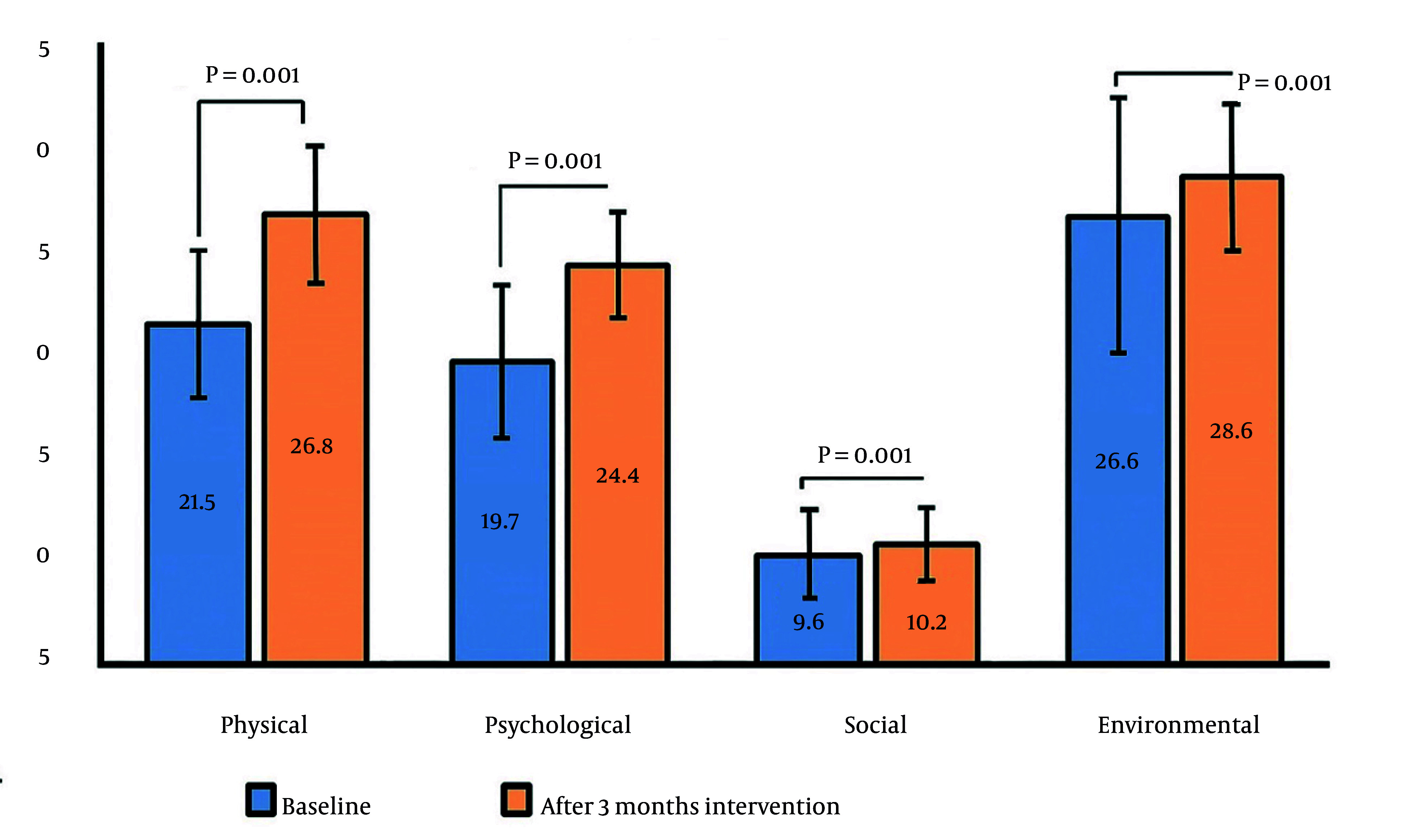
Change in quality-of-life domains after health education intervention

Figure 1 summarizes the changes in QOL domain scores among the 232 participants following the health education intervention. Following the three-month health education intervention, significant improvements were observed across all domains of QOL among the 232 participants. The mean physical health score increased from 21.5 at baseline to 26.8 post-intervention (P = 0.001), while the psychological domain improved from 19.7 to 24.4 (P = 0.001). The social domain showed a modest increase from 9.6 to 10.2 (P = 0.001), and the environmental domain increased from 26.6 to 28.6 (P = 0.001). Overall, these results indicate that structured health education significantly enhanced QOL, with the greatest improvements observed in the physical and psychological domains (Figure 1). These findings indicate that structured health education significantly enhanced patients’ QOL, with the most pronounced gains observed in the physical and psychological domains.

## 5. Discussion

This study evaluated the effects of a structured health education program on the QOL among patients with diabetes. Most of the participants in the study were over 40 years old, with a nearly balanced distribution of males and females. The majority of patients had a BMI greater than 25 kg/m² and had been living with diabetes for more than a decade. These characteristics align with previous research indicating that older adults with long-standing diabetes and higher BMI are at increased risk of diabetes-related complications (10-12).

In terms of treatment, more than half of the patients received combined therapy (oral hypoglycemics plus insulin), reflecting the advanced stage of disease and the complexity of managing glycemic levels in long-term diabetes. This is consistent with evidence suggesting that combination therapy is often required when monotherapy is insufficient to achieve target glycemic control (10, 13).

After the three-month educational intervention, participants showed significant improvements across all QOL domains (Figure 1). The most substantial gains were observed in the physical and psychological domains, supporting prior findings that diabetes self-management education (DSME) enhances both physical health and mental well-being by improving patient knowledge, confidence, and adherence to treatment regimens (12, 13). Social and environmental domains also improved, albeit to a lesser degree, suggesting that structured education may have indirect benefits on social interactions and environmental perception through better self-care practices and healthier lifestyle behaviors (21).

The study results highlight the value of integrating structured health education into routine diabetes management. Fatahi et al. conducted a randomized controlled trial and reported that the self-esteem mean scores of patients in the peer education group (100.36 ± 15.9 vs. 106.87 ± 9.08, P = 0.011) and the virtual education group (100.80 ± 24.72 vs. 116.91 ± 10.67, P = 0.018) significantly increased after the intervention, while no significant difference was observed in the control group (106.87 ± 9.08 vs. 105.60 ± 10.84, P = 0.134), suggesting that education positively impacted the life of diabetic patients (22). Moazeni et al. emphasized from their study that education does help patients to adhere to self-care principles, and addressing the educational needs of both the patient and their family is an essential aspect of care and needs ongoing education to counteract the changes imposed by this chronic disease (23). Programs emphasizing lifestyle modification, medication adherence, and self-monitoring can positively impact both subjective and clinical outcomes (24, 25). Hosseinzadeh et al. recommended that the delivery of high-quality healthcare services is related to patient compliance with doctors' orders, and patients' opinions on healthcare quality are crucial in order to reuse their services in the future. Therefore, the health care educationalists should also focus on the quality of services delivered in the Diabetic department if they want to ensure the potential benefits of their health education on the QOL of diabetic patients (26).

Our findings are in agreement with systematic reviews indicating that individualized educational interventions can improve glycemic control, reduce the risk of complications, and enhance overall QOL in patients with type 2 diabetes (15). Individual treatment goals should take into account the patient's capacity to understand and carry out the treatment regimen, as the risks associated with optimal control of blood sugar may outweigh the benefit of normoglycemia among certain groups of patients, e.g., very young or old age or other coexisting diseases.

The overall improvement in all QOL domains from baseline to 3 months supports evidence that structured diabetes education enhances physical, psychological, and social functioning in chronic diabetes populations (12, 13). Greater gains among participants > 40 years, females, those with higher BMI, and longer diabetes duration reflect findings from previous studies showing that high-risk groups benefit most from targeted education and self-management support (10-12). The modest yet consistent rise in social and environmental scores aligns with reports that improved self-care promotes better social engagement and environmental adaptation (18). Notably, patients without complications demonstrated superior psychological and physical improvements, reinforcing research that early intervention and complication-free status are key predictors of better QOL outcomes (15).

### 5.1. Limitations

Limitations of the present study include small sample size from only two centers and the relatively short follow-up period. Further research involving multiple centers and extended monitoring would provide more insights into the long-term sustainability of QOL improvements and their association with clinical outcomes such as HbA1c and complication rates. Also, its non-randomized design may introduce selection bias and affect the generalizability of the findings. The validity and reliability of the quality-of-life questionnaire have not been previously confirmed for the Saudi Arabian population.

### 5.2. Conclusions

The study demonstrates that structured health education significantly enhances the QOL in patients with diabetes, with the most notable improvements observed in the physical and psychological domains. Integrating such educational interventions into routine diabetes care can empower patients, improve self-management, and contribute to better long-term health outcomes. These findings support the implementation of patient-centered education programs as an essential component of comprehensive diabetes management.

## Data Availability

The dataset presented in the study is available on request from the corresponding author during submission or after publication. The data are not publicly available due to patient privacy.
